# TREC-SAVE: a randomised trial comparing mechanical restraints with use of seclusion for aggressive or violent seriously mentally ill people: study protocol for a randomised controlled trial

**DOI:** 10.1186/1745-6215-12-180

**Published:** 2011-07-20

**Authors:** Gisele Huf, Evandro SF Coutinho, Marco AV Ferreira, Silvana Ferreira, Flavia Mello, Clive E Adams

**Affiliations:** 1 National Institute of Quality Control in Health-Oswaldo Cruz Foundation, Rio de Janeiro, Brazil; 2 University Hospital Clementino Fraga Filho-Federal University of Rio de Janeiro, Rio de Janeiro, Brazil; 3 National School of Public Health-Oswaldo Cruz Foundation, Rio de Janeiro, Brazil; 4 Psychiatric Institute Philippe Pinel, Rio de Janeiro, Brazil; 5 University of Nottingham, Nottingham, UK

## Abstract

**Background:**

Thousands of people whose aggression is thought due to serious mental illness are secluded or restrained every day. Without fair testing these techniques will continue to be used outside of a rigorous evidence base. With such coercive treatment this leaves all concerned vulnerable to abuse and criticism. This paper presents the protocol for a randomised trial comparing seclusion with restraints for people with serious mental illnesses.

**Methods/Design:**

Setting-General psychiatric wards of a large psychiatric hospital in Rio de Janeiro, Brazil. Participants-Anyone aggressive or violent suspected or known to have serious mental illness for whom restriction is felt to be indicated by nursing and medical staff, but also for whom they are unsure whether seclusion or restraint would be indicated. Interventions-The standard care of either strong cotton banding to edge of bed with medications as indicated and close observation or the other standard care of use of a minimally furnished seclusion room but with open but barred windows onto the nursing station. Outcomes-time to restrictions lifted, early change of treatment, additional episodes, adverse effects/events, satisfaction with care during episode. Duration-2 weeks. Identifier: ISRCTN 49454276 http://www.controlled-trials.com/ISRCTN49454276

## Background

The association between mental illness and violent behaviour has been a controversial topic for public health. Violence, however, occurs in about 30% of those who attend psychiatric services for the first time [1]. Violent or agitated patients present a critical risk to themselves, to other patients and to staff, so effective, humane and safe intervention is necessary to prevent injury to everyone involved [2,3]. After de-escalation techniques have failed, restraints, seclusion and/or rapid tranquillisation may be used. Mechanical restraints are often cotton or leather belts tied to the bedside. Seclusion refers to an involuntary confinement of a patient in a special room (locked/unlocked).

### Global use of restraints/seclusion

**Figure 1:**
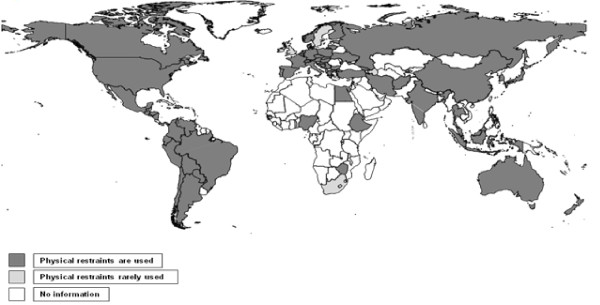
**Countries where restraints are used**.

Mechanical restraints are used in many countries; right across Europe [4-28], the Americas [29-33], the Middle East [34,35], Central [36-38] and South East Asia [39-41], Australasia [42-45] and Africa [46,47] (Figure 1). We have not identified literature describing prevalence of use of seclusion rooms. Where use of restraints is less prevalent [37,48-54] doses of medication tend to be greater and manhandling common.

### Prevalence of use

Survey rates vary (3-50%)[15,25,29,44,55-58], but even the limited epidemiology suggests that hundreds of thousands of people with serious mental illness are in restraints every day. As background for this study we undertook audit of local practice in Psychiatric Institute Philippe Pinel, Rio [59]. This audit, combined with high quality evidence from two proceeding TREC trials [60,61] confirmed that about 25% of people who are aggressive are physically restrained. Subsequent aetiological investigation on the trial dataset showed that, in Rio, risk factors most strongly associated with use of restraints were being young, presenting intense agitation more commonly attributed to substance abuse or diagnoses other than psychosis and arriving in the morning [62].

### Effects and controversy

The safety and clinical effects of mechanical restraints or seclusion in a psychiatry setting are questionable [63]. A Cochrane systematic review found no randomised controlled studies [64]. This comprehensive review methodically searched 12 databases carefully selecting potentially relevant work. Other authoritative reviews also failed to identify other means of objective evaluation [2]. Certainly people are harmed or die in both restraints and seclusion [3,65] but also if they are not used. In addition to reports of physical injury and death, qualitative studies illustrate the negative psychological impact of being restrained [66,67]. Guidelines encourage minimal use and strict regulation of restraint/seclusion but, in practice, this may not occur.

This most coercive part of heath care has avoided any high grade evaluation and therefore the safety of a very large most vulnerable group (and their fellow patients as well as heath care staff) has been neglected.

### Setting

Eighty percent of people across the world live in low or middle income countries and approximately 1-2% of people suffer from severe mental illnesses [14]. There is no evidence that psychiatric emergencies are less prevalent in these countries, therefore, most episodes of aggression for severely mentally ill people take place in these countries. The TREC-SAVE study was designed in collaboration with those working in a busy Brazilian psychiatric care setting to be applicable, at the very least, for everyday local care Psychiatric Institute Philippe Pinel, in Rio de Janeiro, Brazil. It provides a service for 25% of a city of approximately 7 m people and runs a 24 hour psychiatric emergency room and two acute wards and two longer stay wards (70 inpatients, 30 emergencies/day). Research into the evaluation of emergency treatment has been a central part of the activity of these units since 2003 [39,41,60,61] and instigation of new procedures for restraint/seclusion was seen as an opportunity for research once it had been established that the new procedures were not grounded in high grade evidence.

We aim to undertake a pragmatic randomized trial to evaluate, for the first time, the safety, effects and acceptability of two widely applicable ways of restricting aggressive or violent people with serious mental illness; mechanical restraints compared with use of seclusion room.

Secondary aims are to illustrate how objective evaluative techniques can, carefully, thoughtfully, humanely and ethically be applied to this large, neglected, part of health care, and to ensure that the means of evaluation is designed in such a way as to be applicable worldwide.

## Methods/Design

### Size

The aim of TREC-SAVE is to pilot methods, to generate data relevant for sample size calculations and, if possible, clinically useful data regarding whether either procedure is better in terms of clinically measures restriction and perceived treatment success. TREC-SAVE is, in part, a proof of concept study. Two main factors determine the number of people who should be recruited to in order for the trial to provide clear answers. They are the frequency of the investigated event and the size of the effect of treatment. It is important to avoid results that are erroneous. The probability of producing so called 'false-positive' results (type I error-α) and 'false-negative' findings (type II error-β) is minimised by having adequate sample size. However, we have no data at all from trials even to estimate adequate sample size [64] and so we recruited ten people as a very preliminary test of methods and, thereafter, aim to randomise 100 in total. Concerning the primary outcome, 100 patients were needed to give 80% power at a two-sided 5% significant level to detect a reduction in an assumed value of 50% to 30% or of 25% to 10%.

### Ethical considerations

Trials in non-consenting patients are permitted on two conditions: i. no other context exists in which to answer the question; and ii. all trial participants get clear therapeutic benefit from whichever arm they are randomised to. Aggressive patients in a situation of psychiatric emergency are not able to give consent for their participation in a study. We are not proposing to change routine care. All treatment will be open and only people for whom doubt about which care package to give will be eligible. These are packages of care that are given day to day and TREC-SAVE simply adds randomisation to situations where there is clinical doubt and a few additional means of recording safety and acceptability.

We will obtain informed consent from any accompanying relative (Additional file 1, Appendix 6). If there is no accompanying relative we do not plan informed consent at time of randomisation because, by definition, the participant lacks capacity. This is full in accordance with The Helsinki Declaration [68], the European Directive on Clinical Trials [69], the Nuffield Council on Bioethics [70] and the US Food and Drugs Administration guidance [71]. Each guidance states that it is possible to undertake randomisation before gaining consent from the participant once more recovered or their relative once accessible. For example, the Helsinki Declaration reads as follows (section B.29):

"Research involving subjects who are physically or mentally incapable of giving consent, for example, unconscious patients, may be done only if the physical or mental condition that prevents giving informed consent is a necessary characteristic of the research population. In such circumstances the physician should seek informed consent from the legally authorized representative. If no such representative is available and if the research cannot be delayed, the study may proceed without informed consent provided that the specific reasons for involving subjects with a condition that renders them unable to give informed consent have been stated in the research protocol and the study has been approved by a research ethics committee. Consent to remain in the research should be obtained as soon as possible from the subject or a legally authorized representative."

This is a project that has to have the most careful ethical considerations. Current clinical practice on vulnerable [if aggressive] people who are given coercive treatment is necessary but, if carefully considered in the light of the complete lack of research in the area, may be considered barely ethical in itself. The treatment, in the context of a randomised trial tailored to the needs of the services and patient, is, we argue, highly ethical. We have the exacting scrutiny and then support of local Psychiatric Institute Philippe Pinel Ethics Committee which has a track record in protecting the rights of all participants.

### TREC-SAVE is designed to fit into everyday practice

The great majority of clinical trials are explanatory; they are small, short, evaluate rigid care regimens, measure outcomes in ways that are of little clinical value and are difficult to relate to everyday practice [72]. Pragmatic trials, on the other hand, evaluate care that can be used in everyday practice and measure outcomes that are of general concern. This is a pragmatic randomised trial, designed with and for staff and policy makers of Psychiatric Institute Philippe Pinel, Rio de Janeiro, Brazil.

### Randomisation

A fundamental step in such a trial is the randomisation; the distribution of the treatments in a way that is not a function of a clinical decision, but of pure chance. Randomisation will be undertaken in Brazil, blocks of 4 and 6 in random order, within which randomly generated sequences of treatments were generated using the "List Randomizer" option from http://www.random.org/ inputting blocks of 4 and 6 numbers. The sequence for inputting the blocks of 4 or 6 itself being established at random. Allocation will be fully concealed and undertaken by personnel not involved in the clinical interface. Consecutively numbered sealed, fully opaque envelopes, identical in every way to the outside observer will be prepared and each will contain the information regarding trial conduct and directions to either seclude or restrain as per normal care. These envelopes are held in TREC-SAVE boxes on the wards and their order of opening will be checked by the trial researcher.

### TREC-SAVE is blinded for the initial ratings only

**Figure 2:**
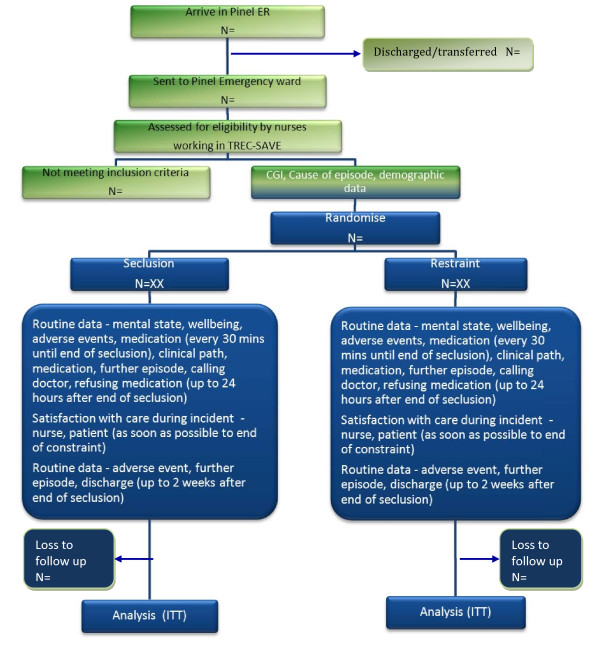
**TREC-SAVE CONSORT diagram**.

It is impossible to blind people to seclusion or restraint, but, in any case, because TREC-SAVE evaluates care in the emergency situation, it is imperative that the doctors and nurses know which intervention is being given. Also, we wish to evaluate *the open giving *of an approach as would happen in everyday care. Full blinding is, therefore, not desirable. The study, however, is blind up until the time that the TREC-SAVE envelope is opened. Therefore, it is crucial that the evaluation of the severity of a person's disturbance and the first impression on the possible cause for the disturbed behaviour are recorded before this envelope is opened (Figure 2).

### Participants

Aggressive/violent mentally ill people admitted to Psychiatric Institute Philippe Pinel, in Rio de Janeiro, Brazil.

#### Inclusion criteria

- Anyone thought to have a serious mental illness admitted to the hospital who has a degree or risk of aggression or violent behaviour that endangers themselves or others; and

- Who is thought by medical and nursing staff to need some form of physical restriction; and

- For whom the medical and nursing staff have doubt as to whether one form of restriction is better than the other.

#### Exclusion criteria

- Anyone for whom either or both packages of care are contraindicated by either medical or nursing staff; or

- Anyone already randomised in this trial.

### Recruitment

According to local audit Psychiatric Institute Philippe Pinel, covers an area that refers to about 600,000 people, and has 90 inpatients every day and sees about 50 people in the emergency rooms. Ten percent of this group are agitated and one quarter need some restraints but we are unclear about what proportion of this group the health care staff have some doubt about whether to use one form of restraint or another. We estimate this to be one in 10 people and therefore it is likely that recruitment will be around 2 persons per week.

### Interventions

1. Use of four point physical restraint (cotton bands) and policy of close recording of mental state, behaviour, wellbeing, in addition to standard use of medication.

2. Use of secure seclusion room and policy of close recording of mental state, behaviour, wellbeing, in addition to standard use of medication.

In the case of Psychiatric Institute Philippe Pinel secure seclusion involves a locked room with minimal bedding but bright and airy with good day light though barred windows with no frame or glass open to the nursing station. There is a simple toilet and sink. The standpoint of care is that both care packages are undertaken wishing the least restriction for the least period of time. The Directorship of the Institution is wishing to increase safety, monitoring and yet decrease restriction of patients and is supportive of this work to ensure that new policies are based on good evidence.

### Procedures

For all eligible patients the administering nursing staff will confirm that they feel that the clinical state of the potential participant still meets eligibility and, if so, take the next sealed envelope in a pre-prepared pack. If they feel that the potential participant does not meet eligibility they will not randomise and proceed with normal clinical care. If possible data on those not entering the study but in need of restraint or seclusion will be recorded (Additional file 1, Appendix 3).

Before opening the envelope the nurse will fill out a Clinical Global Impression [73] regarding the degree of danger and aggression (Additional file 1, Appendix 1, Formulário 1). Inside the envelope will be the allocation to use of restraints, or seclusion and a sticker. The latter will be pasted on the patient's notes to alert staff that this person has been part of the study (and also should not be randomised again). The envelope will be discarded into a specific container. Clinical data will then be recorded (Additional file 1, Appendix 1, Formulário 2). This involves the careful recording of the reasons for seclusion, half hourly monitoring of mental state and wellbeing by nursing staff, hourly monitoring by medical staff and recording of any adverse event or effect as well as the duration of the restriction. At any time the medical or nursing staff will be free to change treatment options.

### Serious events

After trial entry, clinical events are recorded, as usual, in the patients' notes. Complications and adverse events should be managed as usual. A serious unexpected event form (Additional file 1, Appendix 1, Formulário 3) is provided, and will be sent to the TREC-SAVE Co-ordinator as soon it is completed.

### Outcomes

Safe resolution of episode, time in restriction, further episode, amount of medication (in 24 h) and how administered, acceptability to patient/staff, adverse effects-follow up 2 weeks or to discharge if before 2 weeks.

Primary outcomes, requested by the nursing and medical staff of the hospital, are release from restraints or seclusion by 4 hours, and usefulness of allocated treatment as measured by the need or not to change treatment of allocation to another form of constraint.

Patients will be followed for 24 hours after the end of the restriction but routinely collected data on use of medications and further episodes for up to 14 days or until discharge if before. Right after the end of the restriction relevant staff will be asked if they feel the episode was, within the care package, managed satisfactorily and asked to rate this on a simple visual analogue scale. Also, and again right after the end of episode of the restriction, the participant will be asked to rate their satisfaction with the management of the relevant episode and again on simple visual analogue scale.

Other relevant outcomes are: refusal to take oral medication, other episode of restraint/seclusion, need to see the doctor again (Additional file 1, Appendix 8).

### Data collection, entry and analysis

All analysis will be based on groups as randomly allocated; this will be an intention-to-treat analysis. Relative risk, risk difference, number needed to treat and respective 95% confidence intervals will be estimated and for continuous outcomes mean difference will be assessed (Additional file 1, Appendix 8).

### Trial organisation

The TREC-Rio Co-ordinating Group: The co-ordinating centre of the Rio de Janeiro arm is based at Oswaldo Cruz Foundation, Rio de Janeiro, Brazil. The Co-ordinating Group has overall responsibility for the design of the study and is responsible for all aspects of day to day trial administration. The Co-ordinating team is also responsible for preparing reports for the steering committee. Membership: Gisele Huf, Evandro SF Coutinho, Clive Adams.

The TREC-Rio Steering Committee: The overall progress of the trial, adherence to protocol, patient safety and the consideration of new information will be monitored by a scientific and administrative steering committee. At the end of the proposed study period, the Steering Committee will consider the extension of the study, to allow the detection of other important effects. The membership of this committee is: Evandro S.F. Coutinho (Chair), Gisele Huf, Clive E. Adams, Marco A. V. Ferreira, José Lincoln Souza Cruz, Fernando Ramos and Silvana Ferreira.

### Data monitoring

Should recruitment to TREC-SAVE be slow (take more than one year) or very swift (more than 100 in the expected six month recruitment period), an independent data monitoring committee (DMC) will, in confidence, monitor results. Should recruitment to the TREC-SAVE be slow or go beyond 100, interim results will be supplied, in strict confidence to the chair of DMC or as frequently as requested. Meetings of the committee will be arranged periodically as considered appropriate by the chair of the committee. In the light of the interim data, and of any other evidence or advice they wish to seek, the DMC will inform the chair of the steering committee if, in their view: i. there is proof beyond reasonable doubt that for any particular group or subgroup treatment with one or other regiment is clearly indicated or contraindicated or: ii. it is evident that no clear outcome will be obtained. Proof beyond reasonable doubt may be taken as the difference of at least three standard deviations and at least one of the primary outcomes.

The DMC may communicate certain interim analysis to the steering committee or suggest certain protocol changes, but the steering committee will remain responsible for deciding which changes to adopt. The membership of this committee is: Claudio Jose Struchiner (chair), Luiz Antonio Bastos Camacho and Jose Ramón Rodrigues Arras Lopez.

### Funding

No participating centre will directly receive funds for involvement in TREC-Rio. By design, funding for the overall project is minimal. All funding is intramural and everyone involved is undertaking this project as part of their usual funded employment. This support is jointly funded by National Institute of Quality Control in Health-Oswaldo Cruz Foundation, Cochrane Schizophrenia Group, and Federal University of Rio de Janeiro.

### Proposed policy for publication and authorship

Because this area of health care has been so long neglected but is, nevertheless, part of everyday care across the globe, and because the interventions to be studied are inexpensive clinical techniques rather than inaccessible treatments, the impact of this work is likely to be high.

Part of the publication plan is preliminary work already undertaken in preparation for this application on a local survey of the practice of use of restraints for aggressive people with serious mental illness. This has been analysed and published [59]. Full results will be published and disseminated in a variety of fora including academic meetings, the internet and journals. Efforts will also be made to disseminate findings in a usable form though patient advocate group. The Cochrane review will be updated. There will be a collective authorship (the TREC-SAVE Collaborative Team) but also named authors representing that group as for this paper.

### Future research

We are aware that this study will be ground breaking. There are none to precede it. We expect that much will be learnt in how to conduct such a study and the strengths and weaknesses of our simple design. The trial runs well this design can form a template for others. If numerical results are not informative we will have some idea of the power needed for the larger study if we can identify a source of support. Even if the numerical findings were to be clinically meaningful for Rio de Janeiro indiscriminate generalising of the results of such a small trial would not be prudent. Replication will be necessary both locally and in other settings.

## Competing interests

The authors declare that they have no competing interests.

## Authors' contributions

GH drafted the protocol, and worked with the other authors to refine the methods, read and approved the final manuscript.

ESFC helped draft the protocol and worked with the other authors to refine the methods, read and approved the final manuscript.

CEA helped draft the protocol and worked with the other authors to refine the methods.

MAVF supported the development of the idea and the practical detail of methods, read and approved the final manuscript

SF initiated change in Psychiatric Institute Philippe Pinel systems, helped develop and open debate the methods by which evaluation could occur, read and approved the final manuscript.

FM helped with the practical details of methods, read and approved the final manuscript

TREC-SAVE Collaborative Team-supported this work, helped debate and form methods, read and approved the final manuscript.

## Supplementary Material

Additional file 1 **All forms used in the study and dummy outcome tables**. This file contains 8 Appendices. Appendix 1. Forms in Portuguese. Appendix 2. Data transcription forms-translated to English. Appendix 3. Forms for data for those not entering study. Appendix 4. Forms for additional episodes. Appendix 5. Impression of how episode was perceived. Appendix 6. Informed consent-from accompanying relative. Appendix 7. Poster for wards. Appendix 8. Dummy tables. Click here for file
