# Supplementary material for: TREC-SAVE: a randomised trial comparing mechanical restraints with use of seclusion for aggressive or violent seriously mentally ill people: study protocol for a randomised controlled trial
Source: Trials. 2011 Jul 20;12:180. doi: 10.1186/1745-6215-12-180 (PMC3154155; doi:10.1186/1745-6215-12-180)
Supplement: Additional file 1 — All forms used in the study and dummy outcome tables. This file contains 8 Appendices. Appendix 1. Forms in Portuguese. Appendix 2. Data transcription forms-translated to English. Appendix 3. Forms for data for those not entering study. Appendix 4. Forms for additional episodes. Appendix 5. Impression of how episode was perceived. Appendix 6. Informed consent-from accompanying relative. Appendix 7. Poster for wards. Appendix 8. Dummy tables. [file 1745-6215-12-180-S1.DOCX]

**Appendix 1. Original forms in Portuguese**

# INSTITUTO Philippe Pinel

## TERMO DE CONSENTIMENTO LIVRE E ESCLARECIDO

I- DADOS DE INFORMAÇÃO DO SUJEITO DA PESQUISA OU RESPONSÁVEL LEGAL

I.1.NOME DO PACIENTE:_________________________________________________

DOCUMENTO DE IDENTIDADE N.º:____________________ SEXO: M ( ) F ( )

DATA DE NASCIMENTO: ____/____/_____

ENDEREÇO:_______________________________________N.º:_________________

BAIRRO:_________________________CIDADE_______________________________

CEP.:____________________________TEL.:DDD (____)_______________________

I.2.RESPONSÀVEL LEGAL __________________________________________________

NATUREZA (grau de parentesco, tutor, curador,etc)_____________________________

DOCUMENTO DE IDENTIDADE:_______________________ SEXO: M ( ) F ( )

DATA DE NASCIMENTO: ____/____/_____

ENDEREÇO:________________________________________N.º;_________________

BAIRRO:__________________________CIDADE_______________________________

CEP.;_____________________________TEL.: DDD ( )_________________________

II. DADOS SOBRE A PESQUISA CIENTÍFICA

II.1. TÌTULO DO PROJETO DE PESQUISA: **“TREC-SAVE – avaliação de métodos de contenção física em pacientes violentos ou agressivos – ensaio clínico randomisado”**

II.2.PESQUISADOR PRINCIPAL: **Gisele Huf**

CARGO/FUNÇÃO: Médica/Pesquisadora

II.3. AVALIAÇÃO DO RISCO DA PESQUISA:

SEM RISCO ( ) RISCO MÍNIMO ( ) RISCO MÉDIO ( )

RISCO BAIXO ( x ) RISCO MAIOR ( )

II.4.DURAÇÃO DA PESQUISA: **1 ano**

III- REGISTRO DAS EXPLICAÇÕES DO PESQUISADOR AO PACIENTE OU SEU REPRESENTANTE LEGAL SOBRE A PESQUISA,

**Você está recebendo este folheto porque possui algum parente ou necessita de alguma restrição física devido a um comportamento agressivo ou violento. As medidas utilizadas na rotina do hospital são a contenção física e isolamento em quarto adequado, e apesar de utilizadas há muito tempo, e em diversos lugares em todo o mundo, nunca foram avaliadas através de um estudo adequado. Nós lamentamos que isto seja necessário, mas o estudo que estamos conduzindo pretende avaliar o que é melhor para os pacientes no futuro, principalmente em termos de tempo de restrição e conseqüências indesejáveis.**

**A única alteração na rotina que o estudo introduz é um sorteio entre os procedimentos. Se os profissionais da enfermagem têm alguma dúvida sobre o que pode ser melhor para o paciente, se contenção física ou isolamento, ele é candidato a ser incluído na pesquisa. Todos os demais procedimentos que ocorrem na pesquisa são exatamente os mesmos que ocorrem na rotina do atendimento, e a conduta inicialmente designada pode ser alterada a qualquer momento se isto for necessário.**

**A participação no estudo não envolve testes ou exames adicionais, e todos recebem o melhor cuidado disponível. O estudo foi aprovado pelo comitê de ética do Instituto Municipal Philippe Pinel. Se você tiver qualquer dúvida ou desejar informações adicionais, entre em contato com o chefia de enfermagem ou converse com um dos enfermeiros do hospital.**

III.1. Justificativa e os Objetivos da Pesquisa:

OBJETIVOS

**Comparar um protocolo de contenção física com um protocolo de isolamento para pacientes em situação de vilência ou agressividade nos leitos-diagnóstico do Instituto Municipal Philippe Pinel.**

JUSTIFICATIVA

**Avaliar se um dos métodos é melhor em termos de tempo necessário de contenção e segurança para o paciente.**

III.2. Procedimentos que serão utilizados e propósitos, incluindo a identificação dos procedimentos:

**Contenção física no leito com amarras de algodão e isolamento em quarto com janela e banheiro.**

III.3. Desconfortos e riscos esperados :

**A pesquisa certamente envolve riscos e inúmeros desconfortos, mas de forma alguma esses são maiores do que aqueles verificados durante a rotina de atendimento.**

III.4. Benefícios que poderão ser obtidos:

**Uma informação sobre o que é melhor para estes pacientes no futuro.**

IV- ESCLARECIMENTOS DADOS PELO PESQUISADOR SOBRE GARANTIAS DO SUJEITO DA PESQUISA:

**Não se aplica.**

V. INFORMAÇÕES DE NOMES, ENDEREÇOS E TELEFONES DOS RESPONSÁVEIS PELO ACOMPANHAMENTO DA PESQUISA, PARA CONTATO EM CASO DE INTERCORRÊNCIAS CLÍNICAS E REAÇÕES ADVERSAS:

**Nome do investigador: Gisele Huf**

**Instituição Responsável: Instituto Nacional de Controle de Qualidade em Saúde – Fundação Oswaldo Cruz**

**Endereço: Av. Brasil 4365 – Manguinhos Rio de Janeiro RJ Brasil 21040-900**

**Telefone: 3865-5112**

VI. OBSERVAÇÕES COMPLEMENTARES (SE HOUVER):

**Não se aplica.**

VII. CONSENTIMENTO PÓS-ESCLARECIDO:

Declaro que, após convenientemente esclarecido pelo pesquisador e ter entendido o que me foi explicado, consinto em participar do presente Protocolo de Pesquisa.

Rio de Janeiro, ­­_____ de ________________ de 2010.

______________________________________ ____________________________________

Paciente / Representante Legal Investigador Principal / Co-investigador

**Appendix 2. Data transcription forms – translated to English**


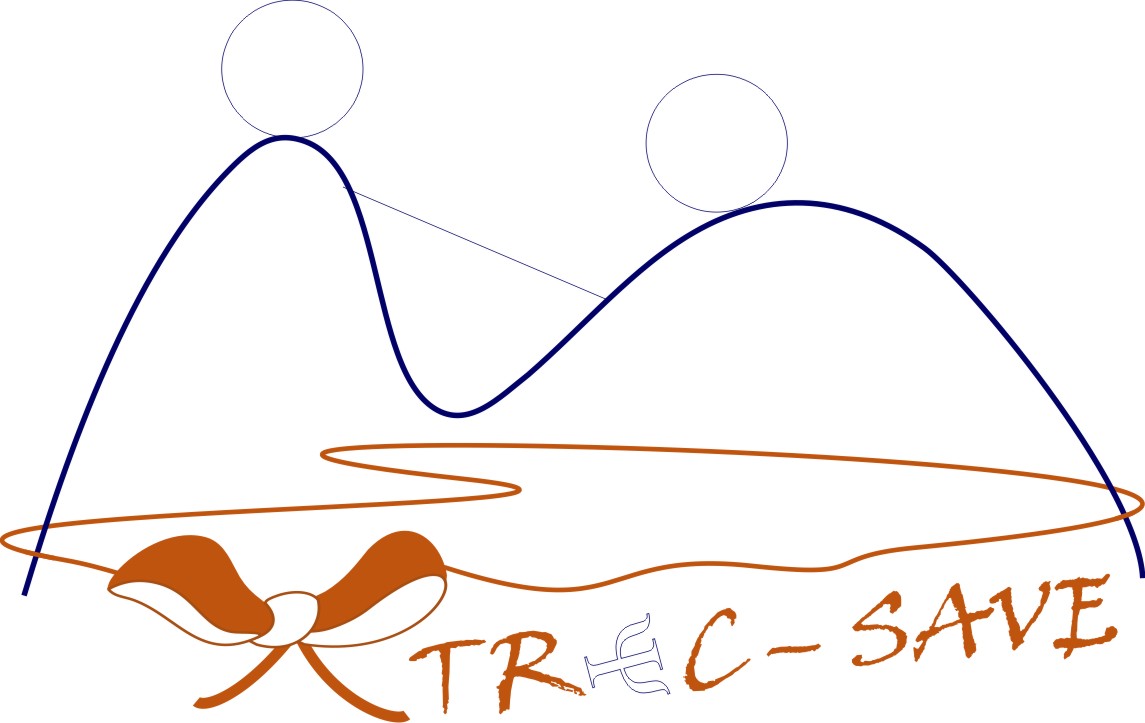


# Form for data transcription Pateints who entered the study

**(YES to all 3 questions – Form 1)**

**Date of collection:** (1) ____ / ____ / _______ (2) ____ / ____ / _______ (3) ____ / ____ / _______

**TREC-SAVE ID number:**

**Hospital record number:

Name of patient:** ____________________________________________________________

**Sex: Male**  Female

**Date of birth:** ____ / ____ / _______ or approximate age**:** ______ years

**Date of admission:** ____ / ____ / _______ **1st Admission?**  Yes No Not sure

 **FORM 1

Date of episode:** ____ / ____ / _______

**Location of intervention:** LDF LDM ER

**What is the severity of this episode?:


What is the cause of this episode?**

Normal

Minimal

Leve

Moderate

Severe

Serious

Amongst the most serious of cases

Psychosis Drugs/Alcohol Unknown Other: ____________________

**N° TREC-SAVE:**


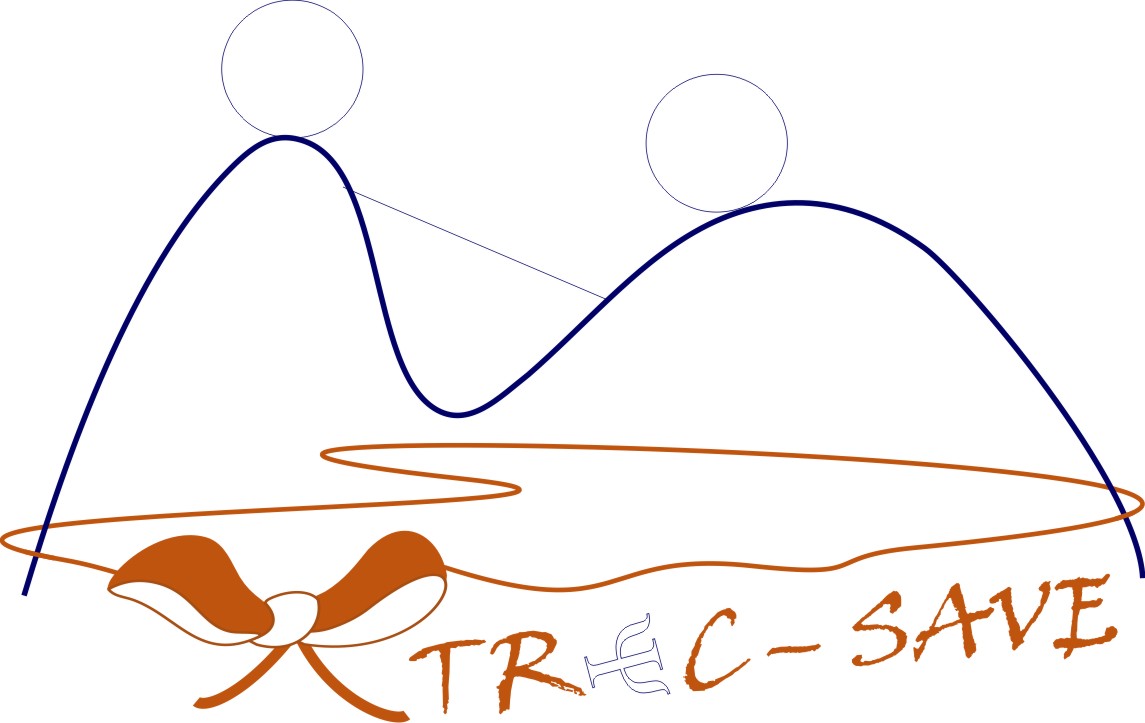


**Procedure allocated** Restraint Isolation

**Time started:** _______ : _______ **Time ended:** _______ : _______

**Person responsible for procedure change:** ________________________ (form **1**)

**There was a need for a change of procedure?** **FORM 2

Procedure alteration:** Restraint > Isolation Isolation > Restraint

**Reason for alteration:** _________________________________________________________

_______________________________________________________________________________

**Time started:** _______ : _______ **Time ended:** _______ : _______

**Person responsible for procedural alteration:** ________________________ (**form 2**)

 **FORM 3
Adverse events recorded in**

**Date of event:** ____ / ____ / _______

**Time:** _______ : _______

Yes

No

Yes No

**the first 24hrs in the**

**bulletin / handbook / notes?

What happened?** _________________________________________________________

_______________________________________________________________________________

**Procedure adopted?:** _________________________________________________________

_______________________________________________________________________________

**Person responsible:** ________________________ (**form 3**)

3/4


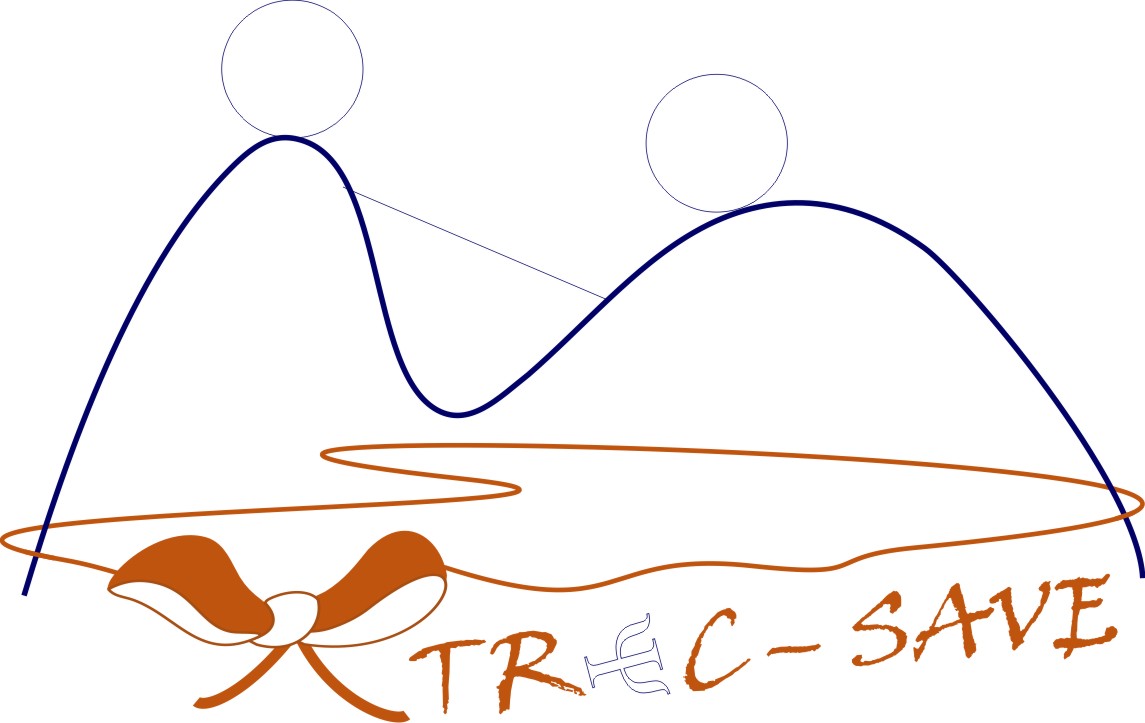


**Other episodes of restraint or isolation? (*):**

Yes How many**?** _______ No

***** If the answer is YES please complete additional form

**24 hour data (handbook / bulletin)**

Yes No

Was there a need to call the doctor in the first 24 hours?

Is there a report of refusal of oral medication in the first 24 hours?

Yes No

#### Medicaitons administered within the first 24 hours after the episode

| Medication | **Dose** | **Route of adm** | **Time** | **Emergancy? (Y/N)** |
| --- | --- | --- | --- | --- |
|  |  |  |  |  |
|  |  |  |  |  |
|  |  |  |  |  |
|  |  |  |  |  |
|  |  |  |  |  |
|  |  |  |  |  |
|  |  |  |  |  |
|  |  |  |  |  |
|  |  |  |  |  |
|  |  |  |  |  |

**14 day data (notes)

Is the pateint still in hospital after 14 days?

If gone:**

Yes No / Data left: ___ / ___ / ______

Discharged

Ran off

Transfer to another psychiatric hospital

Transfter to general hospital

Left of own free will

**Nurses impresson (responsible for form 1)

How do you feel about the way this episode was conducted?**


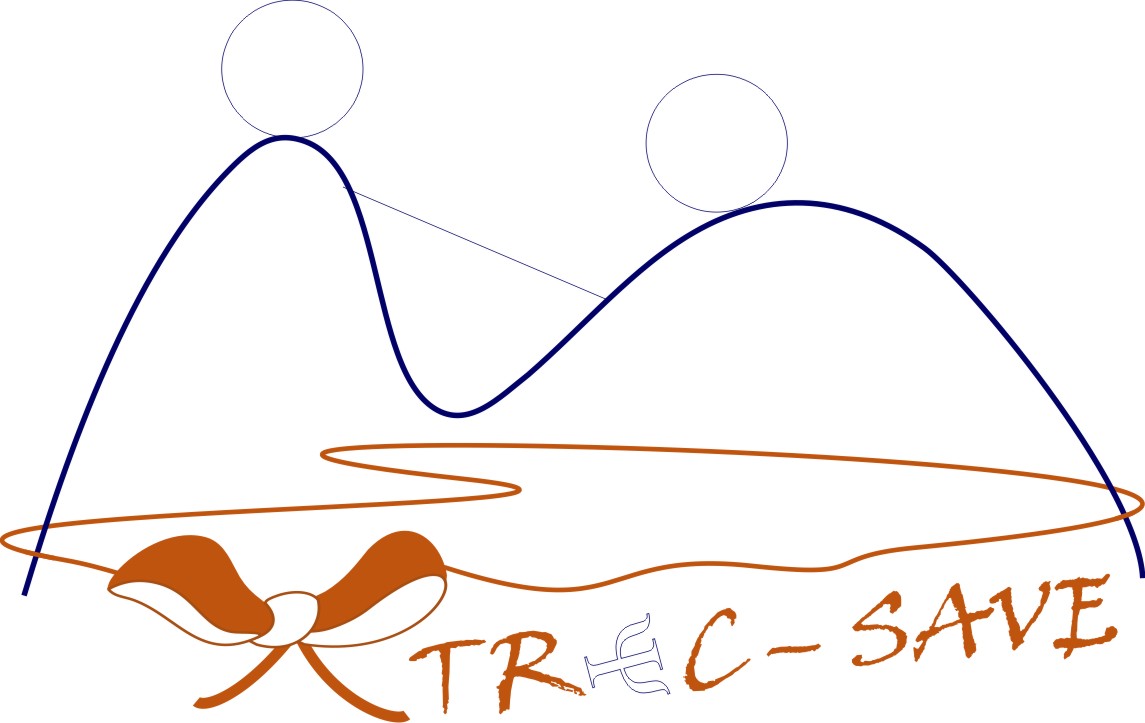


Content

Indifferent

Troubled

Angry

**Pateint’s impression**

**How do you feel about being tied up or being placed alone in the room?**

Content

Indifferent

Troubled

Angry

**Are there any other reasons for containment other than agitation?**_______________________________________________________________________________

_______________________________________________________________________________

**Remarks:**_______________________________________________________________________________

Collector:

**Appendix 3. Forms for data for those not entering study**

# Form for data transcription Patients outside of study

**Date of collection:** (1) ____ / ____ / _______ (2) ____ / ____ / ____ (3) ____ / ____ / _______

**TREC-SAVE ID number:**

**Hospital record number:

Name of patient:** ____________________________________________________________

**Sex: Male**  Female

**Date of birth:** ____ / ____ / _______ or approximate age**:** ______ years

**Date of admission:** ____ / ____ / _______ **1st Admission?**  Yes No Not sure

 **FORM 1

Date of episode:** ____ / ____ / _______

**Location of intervention:** LDF LDM ER

**What is the severity of this episode?:


What is the cause of this episode?**

Normal

Minimal

Leve

Moderate

Severe

Serious

Amongst the most serious of cases

**Do you have any doubt whether this person is better for containment or isolation?**

Psychosis Drugs/Alcohol Unknown Other: ____________________

Yes No

Is the person new to TREC-SAVE?

Yes No

Yes No

Is the isolation room free?

2/3


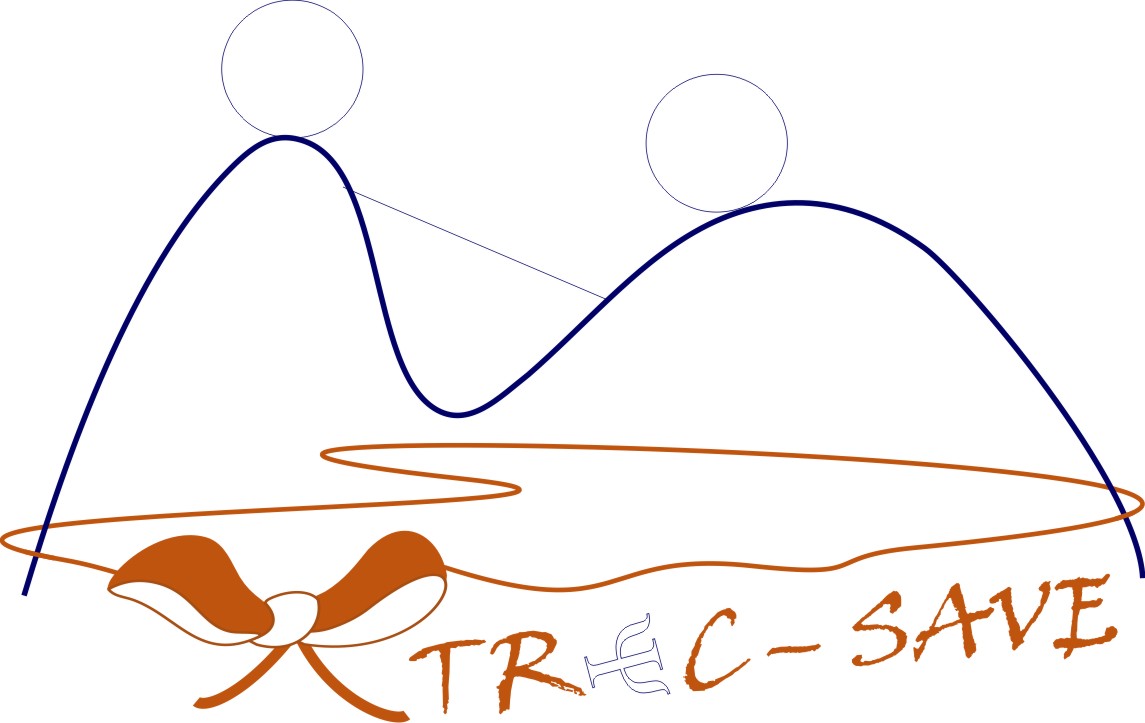


**Procedure:** Restraint Isolation

**Time started:** _______ : _______ **Time ended:** _______ : _______

**Person responsible for procedure change:** ________________________ (form **1**)

**There was a need for a change of procedure?** **FORM 2

Procedure alteration:** Restraint > Isolation Isolation > Restraint

**Reason for alteration: ____**_________________________________________________________

_______________________________________________________________________________

**Time started:** _______ : _______ **Time ended:** _______ : _______

**Person responsible for procedural alteration:** ________________________ (**form 2**)

 **FORM 3

Adverse events recorded in**

**Date of event:** ____ / ____ / _______

**Time:** _______ : _______

Yes

No

Yes No

**the first 24hrs in the**

**bulletin / handbook / notes?

What happened?** _________________________________________________________

_______________________________________________________________________________

**Procedure adopted?:** _________________________________________________________

_______________________________________________________________________________

**Person responsible:** ________________________ (**form 3**)

3/3


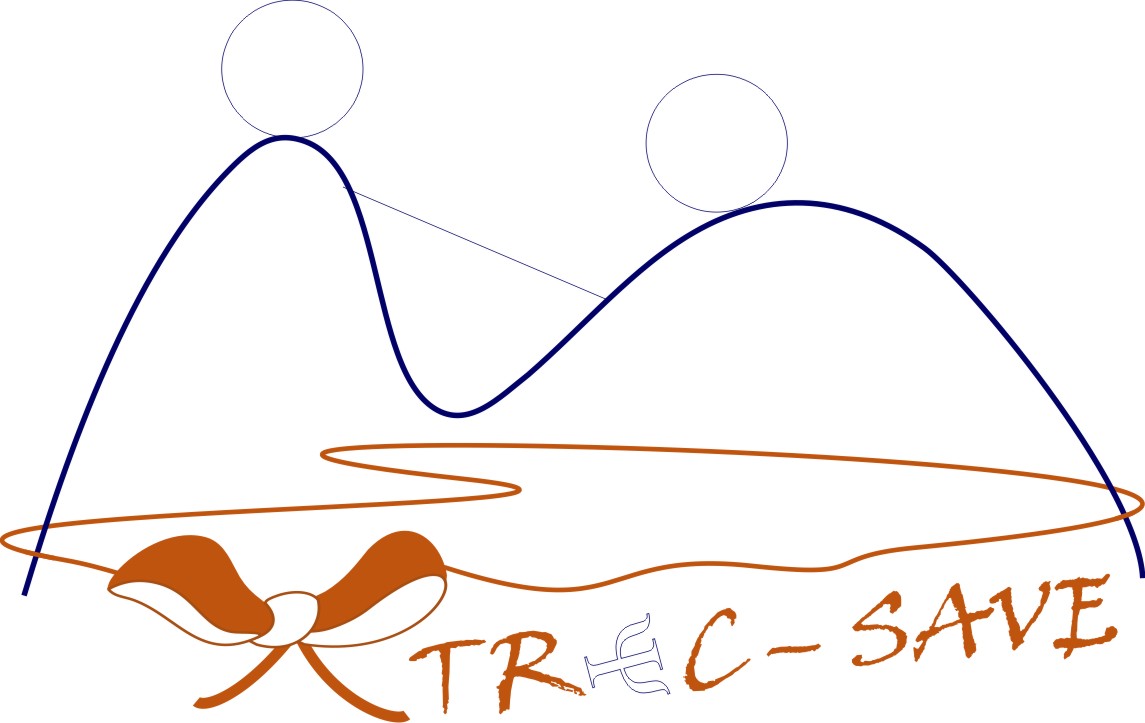


**Other episodes of restraint or isolation? (*):**

Yes How many**?** _______ No

***** If the answer is YES please complete additional form

Collector:

**Dados 24h (prontuário ou livro de ocorrências)** **Data 24 (chart or book of**

**Appendix 4. Forms for additional episodes**

**Collection date:** (1) ____ / ____ / _______

**Hospital record number:**

# Additional form for other episodes For all patients


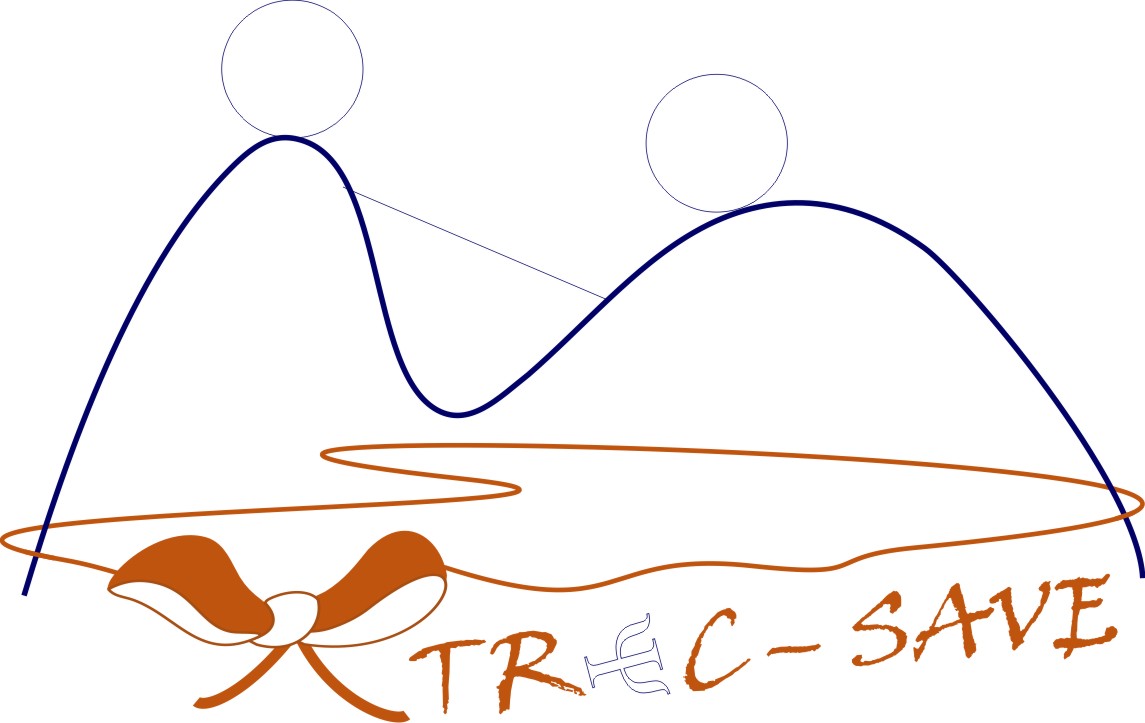


**Name:** ____________________________________________________________

**Date of episode:** ____ / ____ / _______

**Location of intervention:** LDF LDM ER

**What is the severity of this episode?:


What is the cause of this episode?


Procedure chosen:** Restraint Isolation

**Time started:** _______ : _______ **Time ended:** _______ : _______

**Responsible for procedure:** ________________________ (**Form 1**)

**Was there a need for procedural change?

Procedural alteration:** Restraint > isolation Isolation >restraint

**Reason for change:** ________________________________________________________

_______________________________________________________________________________

_______________________________________________________________________________

**Time started:** _______ : _______ **Time ended:** _______ : _______

**Responsible for procedure:** ________________________ (**Form 2**)

Yes No

Psychosis Drugs/Alcohol Unknown Other: ____________________

Normal

Minimal

Leve

Moderate

Severe

Serious

Amongst the most serious cases


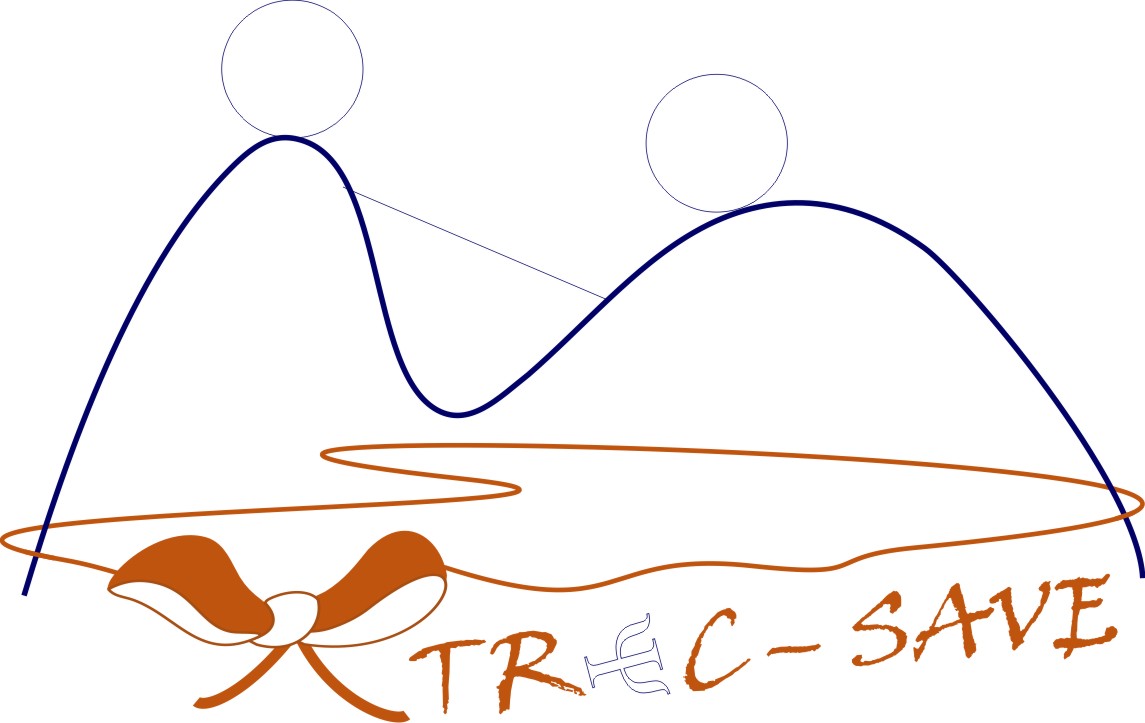


2/2

Collector:

**Appendix 5. Impression of how episode was perceived**


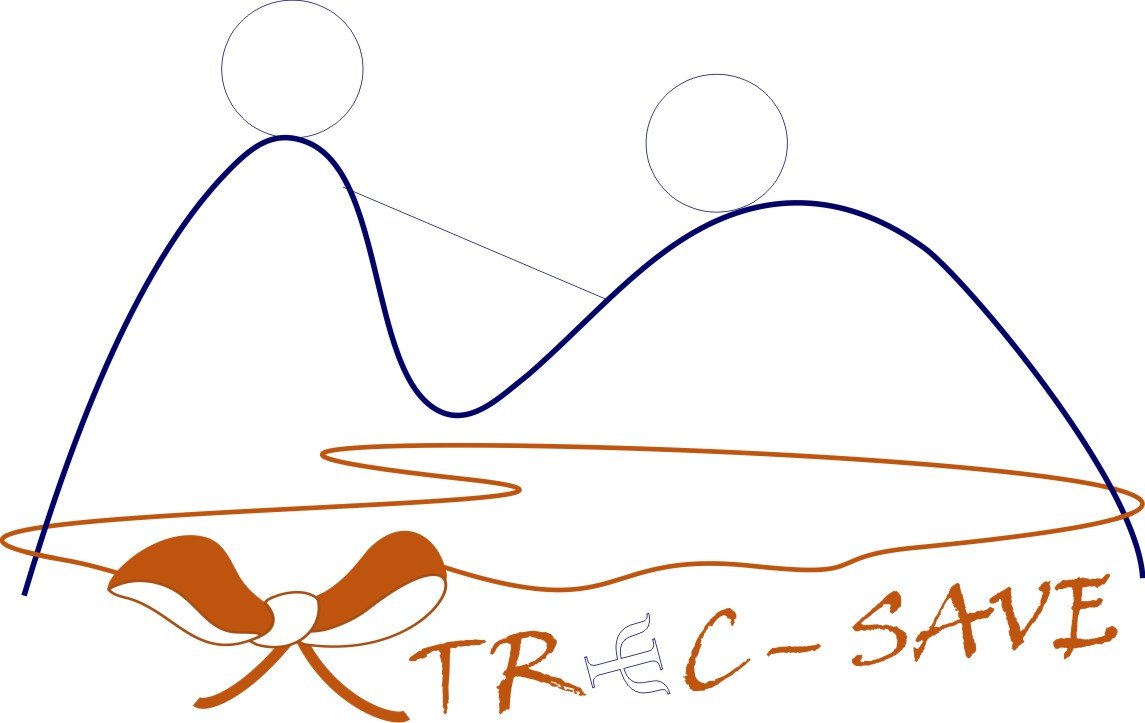


| TREC-SAVE ID |  |
| --- | --- |

# Nurses impression

We know that the use of physical restraint or isolation is difficult for everyone, but now that this episode is over, you could assess how it went?

100% OK

0% OK

Comments

| TREC-SAVE ID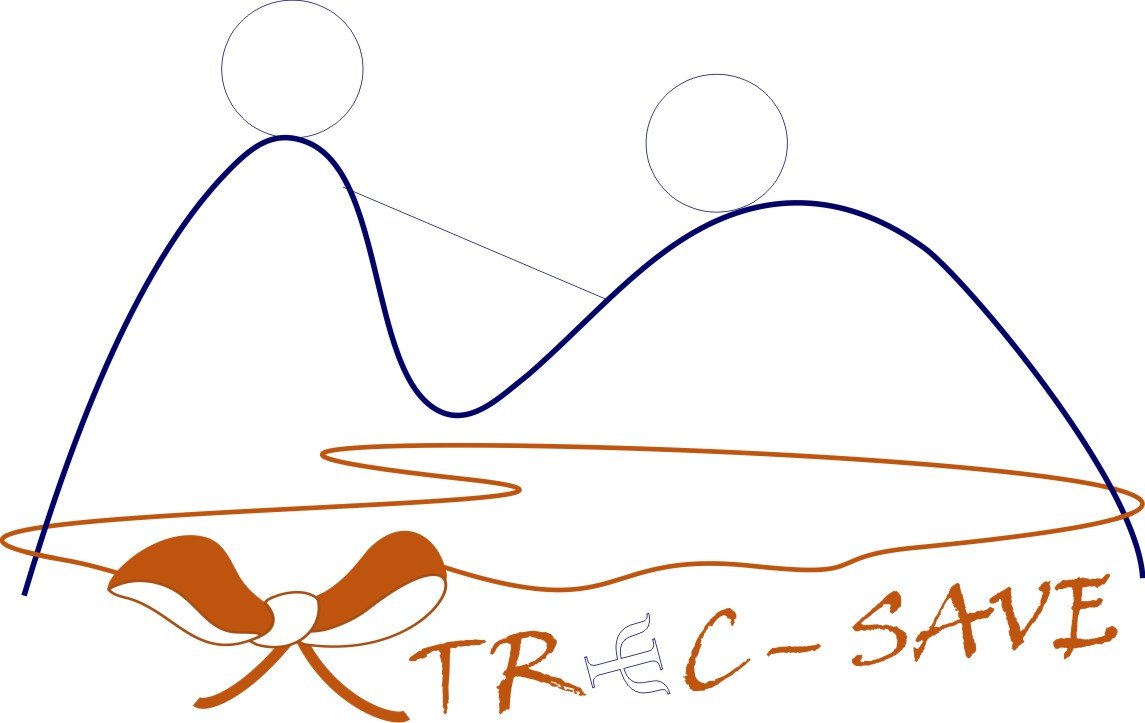 |  |
| --- | --- |

**Patient’s impression**

We know that the use of physical restraint or isolation is difficult for everyone and we regret that this has happened, but we want to improve things in the future and would like to know how you felt about this episode.

Terrible

OK

Comments

INSTITUTE Philippe Pinel
INFORMED CONSENT FORM [English translation]

I-DATA INFORMATION ON THE SUBJECT OF THE RESEARCH

I.1.NOME PATIENT :_________________________________________________
   IDENTITY DOCUMENT N. º:____________________ SEX: M () F ()
   DATE OF BIRTH: ____ / ____ / _____
   ADDRESS :_______________________________________________________
   CITY: _______________________________________________________
  CEP _______________TEL.: (____)_______________________

I.2. LEGAL RESPONSIBLE NAME:__________________________________________________
   NATURE (tutor, curator, etc. )_____________________________
   IDENTITY DOCUMENT :_______________________ SEX: M () F ()
   DATE OF BIRTH: ____ / ____ / _____
   ADDRESS :_________________________________________________________
 CIDADE_______________________________
   CEP___________________ TEL.: DDD ( )_________________________

II. DATA ON SCIENTIFIC RESEARCH

II.1. TITLE OF RESEARCH PROJECT: "TREC-SAVE - evaluation of methods of physical restraints for violent or aggressive patients – randomised clinical trial”

II.2. MAIN INVESTIGATOR: Gisele Huf
    TITLE / POSITION: Physician / Researcher

II.3. RISK ASSESSMENT OF RESEARCH:

NO RISK () MINIMUM RISK () MEDIUM RISK ()
 
LOW RISK (x) GREATER RISK ()

II.4.DURAÇÃO RESEARCH: 1 year

III- EXPLANATION TO THE PATIENT OR LEGAL RESPONSIBLE OF THE RESEARCH

You are receiving this because you have a relative or need to be physically restrained due to an aggressive or violent behavior. The measures used in the routine of the hospital are the physical restraints and seclusion in suitable room, and although they are both used for a long time, and in various places around the world, they have never been evaluated through a proper study. We regret this is necessary, but we are conducting the study to assess what is best for patients in the future, especially in terms of time in restraints and undesirable consequences.

The only change in routine that the study introduces is a lottery between the procedures. If the nursing staff have any doubt on what is best for the patient, whether physical restraints or seclusion, the patient is a candidate to be included in the trial. All other procedures that occur in the trial are exactly the same as occur in routine care, and conduct initially assigned may be changed at any time if needed.

Participation in the study involves no extra tests or exams, and all patients receive the best care available. The study was approved by the ethics committee of the Municipal Institute Philippe Pinel. If you have any questions or wish additional information, please contact the head nurse or talk to one of the nurses at the hospital.

III.1. Rationale and Research Objectives:

OBJECTIVES
To compare a physical restraints protocol with a seclusion protocol for patients in violent or aggressive situation in the emergency wards of the Municipal Institute Philippe Pinel.

BACKGROUND
To evaluate whether any method is better in terms of time required for the restraints and patient safety.

III.2. Procedures that will be used and purposes, including identification of the following:

Physical restraints in bed with cotton ties and seclusion in a room with window and bathroom.
 
III.3. Discomforts and risks expected:
The research certainly involves numerous risks and discomforts, but in no way these are larger than those observed during routine treatment.

III.4. Benefit to be obtained:

Any information about what is best for these patients in the future.

IV- WARRANTIES TO SUBJECT:
Not applicable.

V. INFORMATION OF NAMES, ADDRESSES AND TELEPHONE NUMBERS OF THOSE RESPONSIBLE FOR MONITORING OF THE RESEARCH TO CONTACT IN CASE OF MEDICAL COMPLICATIONS:
Name of researcher: Gisele Huf
Responsible Institution: National Institute for Quality Control in Health - Oswaldo Cruz Foundation
Address: Avenida Brazil 4365 - Manguinhos Rio de Janeiro RJ Brazil 21040-900
Phone: 3865-5112

VI. ADDITIONAL COMMENTS (IF ANY):
Not applicable.

VII. INFORMED CONSENT POST:
I declare that, after properly clarified by the researcher I understood what was explained to me, and I consent to participate in this research protocol.

Rio de Janeiro, _____ ________________ 2010.


____________________________________
              Patient / Legal Responsible

**Appendix 6. Informed consent – from accompanying relative**

___________________________________

Principal Investigator / Co-investigator

**Appendix 7. Poster for wards**


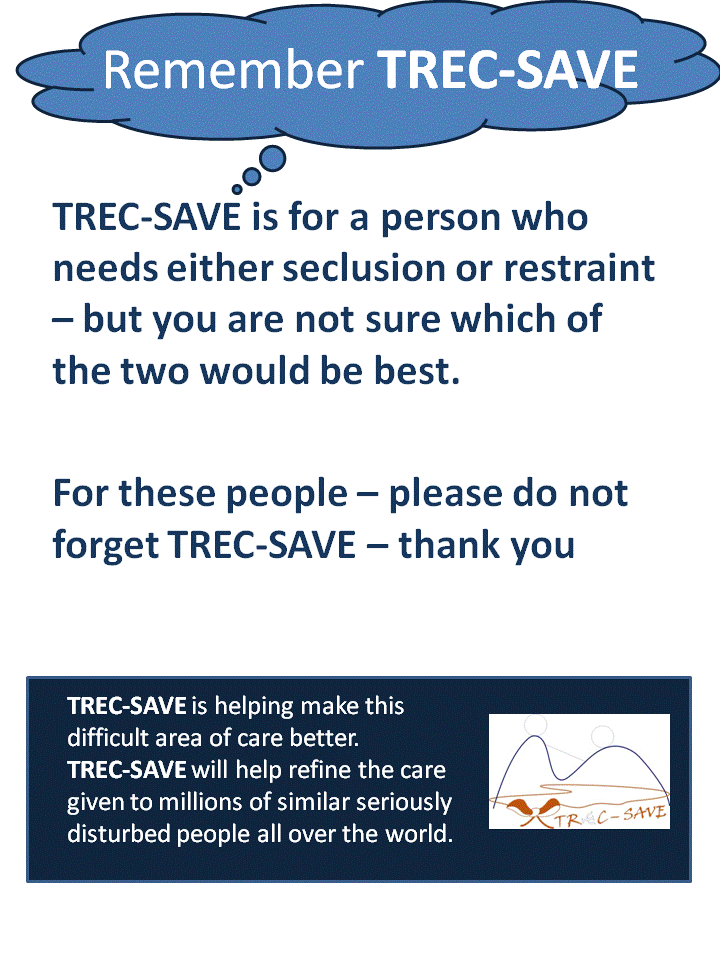


**Appendix 8. Dummy tables**

Dummy table A. Characteristics of patients at trial entry

|  | **Restraints**  **(n = …)** | **Seclusion**  **(n = …)** |
| --- | --- | --- |
| **Mean age (SD)** |  |  |
| **Male / female** |  |  |
| **First psychiatric attendance**  Yes  No  Unknown |  |  |
| ***Severity of disturbance - first impression**  moderately  markedly  severely  among the most extremely disturbed |  |  |
| ***Presumed cause for agitation**  Psychosis (schizophrenia or mania)  Substance abuse  Mental organic (dementia or oligophrenia)  Clinical organic (metabolic, hormones, etc)  Psychological  Unknown |  |  |

* rated blind to treatment of allocation

## Dummy table B. Compliance with the allocated treatment

|  | **Restraints**  **(n = …)** | **Seclusion**  **(n = …)** |
| --- | --- | --- |
| **Allocated treatment** | |  |
| **% continued** |  |  |
| **% changed** |  |  |
| **time of change – mean (SD)** |  |  |
| **Reason for change** |  |  |
| **Time in changed procedure** |  |  |

**Dummy table C. Primary measures of outcome**

|  | **Restraints**  **(n = …)** | **Seclusion**  **(n = …)** |
| --- | --- | --- |
| **Treatment unacceptable / release** | | |
| % Early need to end allocated treatment – within 1 hour |  |  |
| % Not restricted - by 4 hours |  |  |

## Dummy table D. Secondary measures of outcome

|  | **Restraints**  **(n = …)** | **Seclusion**  **(n = …)** |
| --- | --- | --- |
| **Further disturbance / poor compliance** |  |  |
| % Additional episode of aggression needing R/S |  |  |
| % Need to call doctor in first 24 hours |  |  |
| % refusing oral medication |  |  |
| % needing SOS medication |  |  |
| **Release** |  |  |
| Time to release – mean (SD) (total) |  |  |
| % Not restricted by 8, 12, 24 hours |  |  |
| **Medication** |  |  |
| SOS medication  Total load of sedating medication |  |  |
| **Service outcome** |  |  |
| % discharged from psychiatric hospital - by 14 days |  |  |
| % transferred to general hospital within 3 days |  |  |
| **Satisfaction with conduct of episode** |  |  |
| Responsible nurse – % not satisfied, unclear, satisfied |  |  |
| Patient – % not satisfied |  |  |
| **Adverse events** |  |  |
| Any serious event |  |  |
| Specific adverse event |  |  |
|  |  |  |
|  |  |  |
|  |  |  |

Dummy table E. Clinical progress / service outcomes / 2 weeks

|  | **Restraints**  **(n = …)** | **Seclusion**  **(n = …)** |
| --- | --- | --- |
| **Diagnosis** |  |  |
| Diagnosis at 2 weeks or diagnosis at time of discharge, if that was before 2 weeks |  |  |
| **Length of stay – time to discharge / transfer** |  |  |
| % discharged from psychiatric care – by 2 weeks |  |  |
